# Knowledge of Hepatitis C virus vertical transmission and subsequent pregnancy outcome in virus-positive female blood donors

**DOI:** 10.1016/j.bjid.2022.102334

**Published:** 2022-02-16

**Authors:** Hélio Ranes de Menezes Filho, Ludmila Grego Maia, Soraia Mafra Machado, Iasmin Ramos da Silva, Cesar de Almeida-Neto, Ester Cerdeira Sabino, Steven S. Witkin, Maria Cássia Mendes-Corrêa

**Affiliations:** a Universidade Federal de Jataí, Curso de Medicina, Jataí, GO, Brazil; b Universidade Federal de Jataí, Curso de Enfermagem, Jataí, GO, Brazil; c Faculdade de Medicina da Universidade de São Paulo, São Paulo, SP, Brazil; d Universidade Federal de Goiás, Programa de Pós-Graduação em Genética e Biologia Molecular, Goiânia, GO, Brazil; e Fundação Pró-Sangue, Hemocentro de São Paulo. São Paulo, SP, Brazil; f Laboratório de Investigação Médica em Virologia (LIM-52), Faculdade de Medicina da Universidade de São Paulo, São Paulo, SP, Brazil; g Department of Obstetrics and Gynecology, Weill Cornell Medicine, New York, United States of America

**Keywords:** Hepatitis C virus, Pregnancy, Perinatal transmission

## Abstract

**Introduction:**

Hepatitis C virus (HCV) can be vertically transmitted from mother to fetus. We evaluated knowledge about HCV vertical transmission in female blood donors who became pregnant following detection of HCV in their donated blood.

**Methods:**

This was a retrospective descriptive study of females seen at a single blood bank in Sao Paulo, Brazil who were diagnosed with HCV infection in their donated blood. HCV-infected donors who subsequently became pregnant were invited to participate through letters or phone calls. Individuals who agreed to participate were interviewed by questionnaire to evaluate their knowledge on HCV vertical transmission.

**Results:**

Among 282 HCV-positive female blood donors, 69 reported becoming pregnant after their HCV diagnosis in donated blood. While 24 of these women were successful treated for their infection prior to becoming pregnant, 45 (65.2%) were at risk for vertical HCV transmission either because they had never been treated for HCV, were pregnant before treatment or became pregnant after unsuccessful treatment. Of the 59 women who responded to the question of whether they were informed about the risk of HCV vertical transmission, 58 (98.3%) reported never receiving this information either after obtaining their blood donation results or during their pregnancy.

**Conclusion:**

The lack of knowledge of HCV-infected women on the possibility for mother-to-child transmission of this virus highlights the critical need to improve communication about pregnancy-related risks between health professionals and HCV-infected women of childbearing age.

## Introduction

HCV infection has a global incidence rate of 23.7 per 100,000 inhabitants, according to the World Health Organization (WHO).^1^ Most HCV infections are contracted through direct blood contact or by the use of blood-contaminated needles. Vertical transmission of HCV from pregnant mother to her fetus is also possible and is associated with alterations in fetal development, premature delivery and spontaneous abortion, in addition to other obstetric comorbidities.^2^, ^3^, ^4^ The rate of HCV vertical transmission is reported to be 5.8% in children born to women positive for HCV RNA and without co-infection with HIV, and 10.8% in HCV-HIV co-infected women.^4^^,^^5^ In Brazil, the few studies on this subject have estimated a 0.9-1.5% prevalence of HCV in pregnant women and an 0.2% rate of vertical transmission.^6^^,^^7^

In contrast to hepatitis B or HIV infections, testing pregnant women for HCV is not always routine and typically may be restricted to vulnerable populations such as intravenous drug users, those with HIV infection, or if engaging in risky sexual behaviors.^8^ In addition, many clinicians as well as the general public may lack sufficient knowledge of HCV infection and its consequences for pregnancy.

The aim of the present study was to assess the level of knowledge regarding the possibility of HCV vertical transmission among women who were initially diagnosed with HCV infection during blood donation at a blood bank and who subsequently became pregnant.

## Materials and methods

This is a descriptive, retrospective study conducted among a cohort of female blood donors identified as being HCV-positive at the time of their donations at Fundação Pró Sangue/Hemocentro of São Paulo (FPS) from 1994-2012. Details of this cohort have been described previously.^9^ Participants were screened for HCV infection by enzyme-linked immunosorbent assay (ELISA) and RIBA HCV 3.0 strip immunoblot assay. Infection was confirmed by real-time polymerase chain reaction (PCR) (Abbott Molecular, Des Plaines, IL).^10^ This study was approved in December 2011 by the Ethics Committee for Research Project Analysis (Cappesq), protocol n. 8583. All subjects provided informed written consent.

HCV-positive female blood donors identified in this cohort who subsequently became pregnant were contacted through letters, phone calls or emails. Women who agreed to participate were interviewed by questionnaire seeking the following information: sociodemographic data, risk factors for HCV transmission (previous drug use or blood transfusion) and data related to awareness of HCV infection (access to laboratory tests and medical services, compliance with clinical follow-up, and drug therapy). Specific questions related to vertical transmission were also included: if their pregnancy was before or after the diagnosis of HCV, number of pregnancies, occurrence of HCV vertical transmission after blood donation and whether HCV vertical transmission was discussed with blood bank personnel or with their obstetrician after becoming pregnant.

Data were analyzed by the STATA program, version 14.0. Initially, the *Shapiro-Wilk* test was performed to verify normality of quantitative variables.^11^ Following this, a descriptive analysis was performed. Qualitative variables were described as absolute and relative frequency while quantitative variables are presented as mean and standard deviation (SD).

## Results

One-thousand and forty-eight HCV-positive female blood donors were identified. Seven hundred and thirty-six (70.2%) were not localized, 5 (0.5%) refused to participate in the study, 25 (2.4%) had died, and 282 (26.9%) women agreed to participate and answered the questionnaire.

Among the 282 women, 69 (24.5%) reported becoming pregnant after their HCV diagnosis. The mean age of these 69 women was 47.5 years (SD: 11.3; 95% CI: 44.8-50.2) with a median of 48 years (range 28 to 68) at the time of study participation. Table 1 presents the sociodemographic profile and risk factors for HCV transmission for the study group. In terms of race, the population was almost equally divided between white (50.7%) and non-white (49.3), 39.1% had ≤ 9 years of schooling, 44.9% completed 10 to 12 years, 13% had more than 12 years, and 2.9% did not respond to this question. Regarding exposure to HCV, 46.4% had a history of blood transfusion while 4.3% and 1.3% of the women reported use of injected or inhaled drugs, respectively. No risk factor for exposure to HCV was identified by 36.2% of participants.

**Table 1:** Characteristics of the study population (*n* = 69).

Variable	No. positive (%)
Race	
White	35 (50.7%)
Non-White	34 (49.3%)
Education (years)	
≤ 9	27 (39.1%)
10-12	31 (44.9%)
>12	9 (13.0)
Unknown	2 (2.9%)
HCV exposure	
Blood transfusion	32 (46.4%)
Intravenous drug usage	3 (4.3%)
Inhaled drug usage	9 (13.0%)
Unknown	25 (36.2%)

HCV: Hepatitis C virus

The timing of pregnancy in relation to HCV treatment is shown in Table 2.

**Table 2:** Pregnancy history of study population (*n* = 69).

Time of pregnancy in relation to HCV	No. women (%)
No treatment for HCV	27 (39.1%)
After spontaneous elimination of HCV	13 (18.8%)
Before treatment for HCV	10 (14.5%)
After unsuccessful treatment	8 (11.6%)
Unknown	7 (10.1%)
After successful treatment	4 (5.8%)

HCV: Hepatitis C virus.

Twenty-seven (39.1%) of the women were never treated for HCV infection, 13 (18.8%) became pregnant after successful spontaneous HCV clearance, 10 (14.5%) became pregnant before treatment for HCV, 8 (11.6%) became pregnant after treatment but with an unsuccessful anti-HCV response and 4 (5.8%) became pregnant after curative HCV treatment. The timing of pregnancy in relation to HCV treatment was not reported by seven (10.1%) women.

Out of the 59 women who responded the question about being informed of the risk of HCV vertical transmission during pregnancy, 58 (98.3%) reported that they had never received this information. The remaining 10 women could not recall whether they had been told of this possibility. Among the 45 women at risk for vertical transmission of HCV, one (2.2%) reported that the newborn was HCV-positive after delivery. The mother of this baby denied having been informed about the risk of HCV vertical transmission. None of the 24 babies born to women who successfully cleared their HCV infection were vertically infected by HCV.

## Discussion

Our study revealed that there was a glaring deficiency in conveying information about the possibility of HCV vertical transmission, either at the time of their diagnosis or subsequently during pre-natal counseling, to women who tested positive for this virus at a blood bank screening and who subsequently became pregnant.

Mean age, ethnicity and educational level analyzed in this study are consistent with the characteristics already described in other series of chronic HCV-infected individuals in Brazil.^12^^,^^13^ Also exposure factors for HCV transmission reported among the infected women reflected the most prevalent factors reported to the Brazilian health system in the analyzed period.^13^, ^14^, ^15^

The educational level of the study sample does not seem to have influenced the observed results, as these patients consisted of women with different levels of education. Also, the average educational level of the population analyzed was nine years of schooling, which reflects the average educational level of the Brazilian population at the time.^16^

Recommendations for HCV testing in pregnancy differ among organizations. According to the American Centers for Disease Control and Prevention, testing for HCV is recommended for all pregnant women in every pregnancy including women under the age of 18, except in places where the prevalence of HCV infection is less than 0.1%.^17^ In Brazil, the Ministry of Health recommends screening for HCV only in situations with a high risk of transmission, such as in intravenous drug users and recipients of blood transfusions.^18^ Conversely, the Brazilian Federation of Gynecology and Obstetrics Association (Febrasgo) recommends that all pregnant women should be screened for anti-HCV antibodies.^19^ Despite the inconsistency that exists among organizations worldwide, numerous studies have encouraged the implementation of HCV screening during pregnancy to prevent long-term complications.^18^, ^19^, ^20^, ^21^, ^22^.

With the recent availability of new highly effective drugs to eliminate HCV, the identification and treatment of HCV-infected women of childbearing age is a legitimate strategy with the potential to eliminate HCV vertical transmission. In addition, the possibility of universal HCV screening in pregnant women should be considered. When the mother is HCV-positive she can be referred for treatment after delivery and her newborn followed closely to rule out vertical infection.^8^^,^^17^^,^^23^^,^^24^ The cost-benefit ratio of screening in these cases needs to be assessed, keeping in mind the availability of successful treatment to eliminate the virus. Ending the chain of transmission is more advantageous than long-term follow-up of infected children.^17^^,^^23^

Studies on HCV vertical transmission are still scarce, as well as evaluations of knowledge of HCV and its consequences in both infected women and in the general population. This gap in knowledge highlights the urgent need to intensify public health policies to educate the public with the goal of reducing the risk of infection and transmission.^9^^,^^16^ This should not be neglected by health teams.^25^ The WHO has adopted a global strategy to disseminate HCV-related information. Dynamic approaches to conveying the risk of being HCV-positive as a component of general health education and geared toward specific vulnerable populations are being generated.^1^

Limitations of our study should be acknowledged. The small number of cases evaluated may have resulted in a skewed analysis thus not representative of the overall women population. A retrospective investigation based on interviewing the women, in some cases, years after the initial detection of HCV in donated blood or during the pregnancy could have compromised the accuracy of information recall in some cases. Lastly, completion of the study 7-8 years ago may lead one to question the relevance of the data to the present time. However, there is no data to suggest that protocols have been modified over the subsequent time period. We strongly suggest that the study remains valid today and, most importantly, clearly highlights the need for a modified approach to educate individuals to the consequences of infections diagnosed through blood donation.

## Conclusions

The lack of information concerning the possibility of HCV vertical transmission among female blood donors suggests that, in addition to screening for HCV in this population, it is necessary to improve communication between blood center professionals and women at reproductive age, emphasizing the possibility of HCV transmission during gestation and the and its adverse consequences.

## Conflicts of interest

None.
